# Multiple myeloma bone disease models with integrated bone marrow adipose tissue for advancing personalized medicine

**DOI:** 10.3389/fonc.2026.1982576

**Published:** 2026-10-28

**Authors:** Drenka Trivanović, Slavko Mojsilović, Nikola Bogosavljević, Marko Vujačić, Alanna C. Green, Aleksandra Jauković

**Affiliations:** 1 Group for Hematology and Stem Cells, Institute for Medical Research, National Institute of Republic of Serbia, University of Belgrade, Belgrade, Serbia; 2 Institute of Orthopedics Banjica, Belgrade, Serbia; 3 Faculty of Medicine, University of Belgrade, Belgrade, Serbia; 4 Mellanby Centre for Musculoskeletal Research and School of Medicine and Population Health, Faculty of Health, University of Sheffield, Sheffield, United Kingdom

**Keywords:** bone, bone marrow adipose tissue, multiple myeloma, organoid, stem cells

## Abstract

Multiple myeloma (MM) is an incurable hematological cancer of monoclonal plasma cells, where patients commonly develop osteolytic bone disease. The pathogenesis of myeloma is known to be strongly dependent on the bone marrow (BM) microenvironment, but despite this, successful therapies targeting the niche constituents are lacking. Increased body weight and obesity are associated with poor prognosis and disease outcome, making adipose tissue an important regulatory factor in myeloma. Bone marrow adipose tissue (BMAT) has been recognized as a unique adipose depot, with strong implications in MM bone disease. In this review, we discuss the development of advanced adipose tissue models, including BMAT, and their utility for the design of precise and potentially personalized myeloma therapies. Compared to other adipose models, BMAT models require a complex composition to mimic the BM niche, including BM adipocytes, extracellular matrix, immune cells, bone cells, distinct stem/progenitor cell populations plus myeloma cells. In this sense, BMAT-permissive multicellular 3D models, such as bone and BM organoids, could have significant potential to recapitulate the MM niche. Although advanced 3D models based on induced pluripotent stem cells recreate the mesenchymal–vascular–hematopoietic tissues and BM microenvironment, adult BM recapitulation is still challenging. Incorporating patient-derived cells such as myeloma cells and BM adipocytes into these models will improve the accuracy of determining drug responses, disease progression, and patient-specific therapies, particularly in relapsed and/or refractory MM. Altogether, advanced patient-derived 3D model systems provide a more reliable experimental platform for mechanistic and translational studies across the whole BM and bone ecosystem.

## 1 Introduction

Multiple myeloma (MM) is a malignant hematological disorder characterized by clonal expansion of neoplastic plasma cells (PCs) within the bone marrow (BM), accompanied by overproduction of monoclonal immunoglobulins and severe osteolytic bone destruction (1, 2). It is the second most prevalent hematologic malignancy (following non-Hodgkin lymphoma), representing more than 10% of all hematological malignancies and around 1% of all cancers diagnosed worldwide (2). The Global Burden of Disease Study 2021 reported 149,000 new cases and 116,360 deaths annually, with the steepest projected increase in low and middle sociodemographic index regions, reflecting disease control challenges in the lower-income countries (3, 4). The disease affects older people, with median age at diagnosis approximately 65-70, and is more common in males (ratio ~1.5:1) (3). Known risk factors associated with MM also include genetic predisposition, environmental factors such as radiation and certain chemicals, as well as lifestyle factors, including high body mass index (BMI) (4). Availability of advanced therapeutic approaches in the past decades, including autologous stem cell transplantation (ASCT) and the novel therapeutic agents such as proteasome inhibitors and immunomodulatory drugs, as well as the advent of new immunotherapeutic options, like monoclonal antibodies and chimeric antigen receptor T-cell (CAR-T) therapy, have extended patient survival beyond 10 years in transplant-eligible patients (2). Still, MM remains incurable in the vast majority of patients.

The defining pathological hallmark of MM is the uncontrolled clonal expansion of neoplastic PCs that produce monoclonal immunoglobulins (M-protein) detectable in serum and/or urine. According to the revised International Myeloma Working Group (IMWG) criteria, MM diagnosis requires ≥10% clonal PCs in the BM or a biopsy-proven plasmacytoma, together with at least one myeloma-defining event, i.e., CRAB features (hypercalcemia, renal insufficiency, anemia, and bone lesions) or biomarkers of malignancy (≥60% clonal BM PCs, an involved/uninvolved serum free light chain ratio ≥100, or more than one focal lesion on MRI) (2). Increased risk of infections is another hallmark of MM and one of the leading causes of death in MM patients. This is attributed to multiple factors, primarily reduced polyclonal immunoglobulin production (immunoparesis), cytopenias, T, B, and NK cell disfunction, as well as therapy-induced immunosuppression (5).

MM usually evolves from asymptomatic premalignant stages: monoclonal gammopathy of undetermined significance (MGUS) and smoldering multiple myeloma (SMM) (2). In MM, clonal plasmacytosis is usually confined to the BM. Its spread to extramedullary sites (plasmacytomas) or to the peripheral blood (plasma cell leukemia) indicates particularly aggressive disease. Risk stratification is fundamental to accurate prognosis and appropriate therapy selection. Survival can range from a few months to over a decade depending on underlying biology and treatment response. High genomic and clinical heterogeneity of MM has led to multiple revisions of risk stratification systems. At cytogenetic level, abnormalities that confer poor prognosis are t (4, 6), t (6, 7), t (6, 8), del(17p), chromosome 1 abnormalities, and p53 mutation (2, 9). Additional markers of high-risk MM include indicators of patient status and tumor burden, like high level of serum beta-2-microglobulin (β2M), circulating clonal PCs or high frequency of cancer clonal fraction in BM, and elevated serum lactate dehydrogenase (LDH) level (2, 9).

The cytogenetic classification only superficially display true molecular heterogeneity of MM, as individual tumors can harbor more than 400 somatic mutations, which can negatively impact survival (10). Moreover, a comprehensive multi-omics study by Ramberger et al. identified subtype-specific (phospho)proteomic signatures of aggressive plasma cell malignancies, providing a basis for refined risk stratification and novel therapeutic targeting (11).

Bone damage is the most prevalent and debilitating complication of MM, affecting 80–90% of patients during the disease course (12–14). It manifests as osteolytic lesions, pathological fractures, spinal compression and generalized osteopenia, leaving patients with severe bone pain, reduced mobility and quality of life. Unlike metastases from solid tumors, myeloma bone lesions exhibit absent or negligible new bone formation. This uncoupling of osteoclastic resorption from osteoblastic formation is a hallmark of MM bone disease, producing osteolytic lesions that rarely heal, even during prolonged remission (15). At the core of this complex pathophysiologic phenomenon are interactions between MM cells with the BM niche, including BM (mesenchymal) stromal cells (BMSCs), osteoclasts, osteoblasts, and osteocytes (12–14, 16) (**Figure 1A**). Through very late antigen-4(VLA-4)/vascular cell adhesion molecule-1 (VCAM-1)-mediated contact myeloma cells stimulate BMSCs to produce macrophage colony-stimulating factor (M-CSF) and receptor activator of nuclear factor kappa-В ligand (RANKL), the key mediators of OC differentiation and activation. Simultaneously, BMSCs stimulate myeloma cells to secrete other OC-activating factors, primarily macrophage inflammatory protein (MIP)-1α (chemokine (C-C motif) ligand 3, CCL3), interleukin (IL)-6, and tumor necrosis factor (TNF)-α. At the same time, myeloma cells inhibit BMSC production of osteoprotegerin (OPG), the soluble decoy receptor for RANKL, thereby shifting the receptor activator of nuclear factor kappa-B ligand (RANKL)/osteoprotegerin (OPG) ratio in favor of osteoclastogenesis. Bone resorption by activated osteoclasts in turn releases growth factors, transforming growth factor (TGF)-β and insulin-like growth factor (IGF)-1, sequestered in bone matrix, further amplifying proliferation of MM cells. This establishes a self-reinforcing vicious cycle of tumor expansion and bone destruction (12–16). On the other hand, myeloma cells suppress osteoblastogenesis through expression of soluble factors, like Dickkopf-1 and osteocyte-derived sclerostin, that block RUNX-related transcription factor 2 (Runx2) and Wnt/β-catenin signaling in BMSCs, the key molecular pathways regulating OB differentiation (6, 7, 12, 15, 17). Moreover, malignant PCs promote the stability of adipocyte transcription factor PPARγ2, shifting the differentiation of BMSCs toward adipogenesis (18). Furthermore, myeloma cells induce increased production of RANKL, and sclerostin, while reducing OPG secretion by osteocytes. Malignant PCs ultimately lead to osteocyte apoptosis, disrupting the canalicular network that coordinates skeletal homeostasis (12). Thus, we can conclude that MM actively remodels the BM, as well as bone tissue, thereby establishing new relationships at the cellular and extracellular level. First of all, malignant PCs affect the lineage commitment of stem (primarily BMSCs), but also hematopoietic and endothelial progenitors of the BM. This review integrates three lines of evidence on bone marrow adipose tissue (BMAT) in MM (**Table 1**): clinical studies, which associate marrow adiposity with MM risk and progression but cannot establish causality; *in vitro* and murine studies, which identify mechanisms of BM adipocytes (BMAd)–myeloma crosstalk requiring validation in humans; and emerging 3D models, such as BM organoids (BMOs), which may bridge these findings in a patient-specific context.

**Figure 1:**
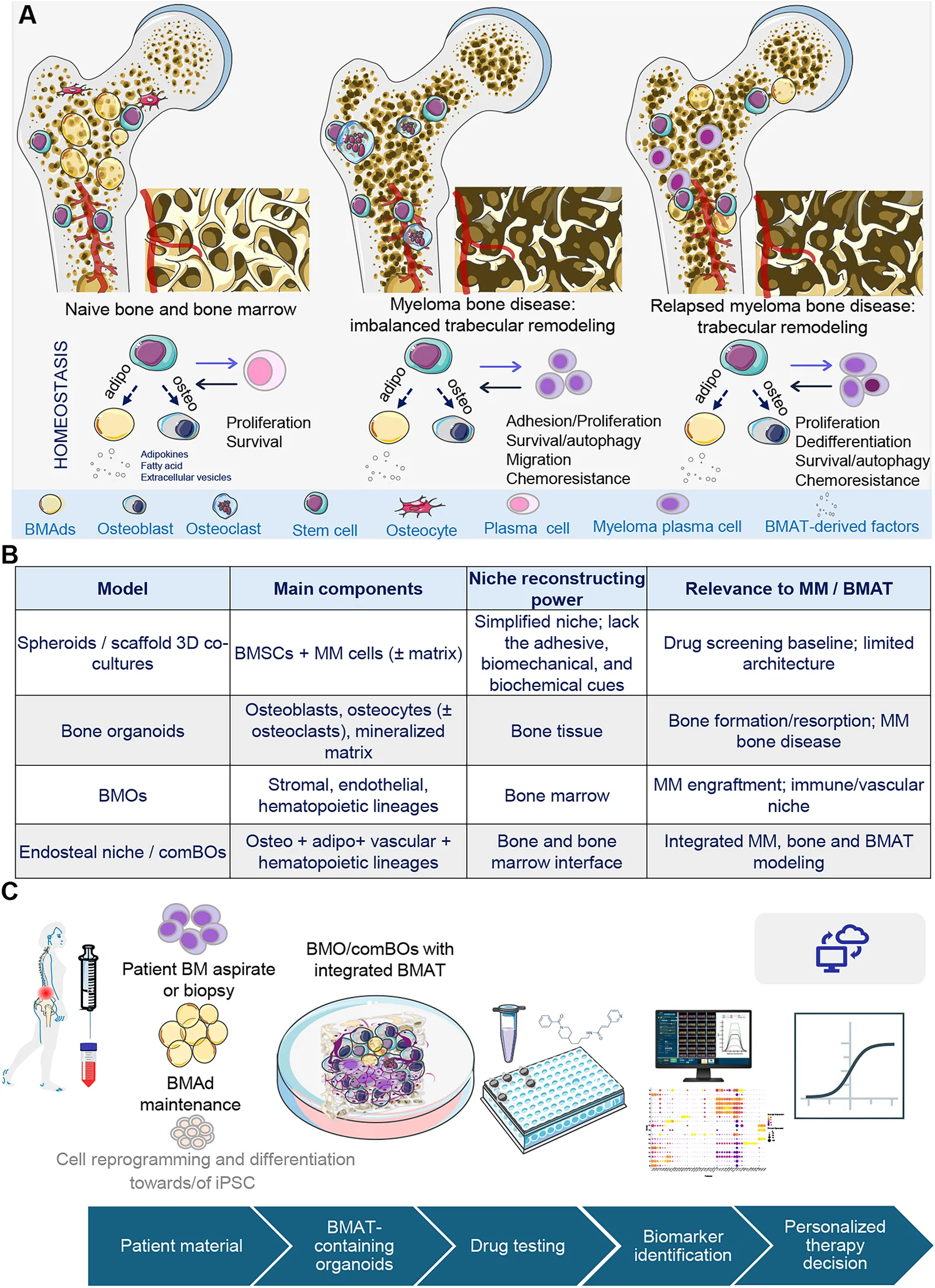
Bone marrow adipocytes in the myeloma niche: from pathophysiology to patient-specific organoid models. **(A)** Diseased bone and marrow environment facilitate relapse of multiple myeloma and the evolution of a heterogeneous and drug-resistant MM cell clones. The presence of multiple myeloma (MM) cells leads to a decrease in bone formation mediated by the differentiation of BMSCs into osteoblasts. In the next phase, osteoclast-mediated bone resorption is increased by MM cells as well as bone lining cells, while osteocytes undergo apoptosis. MM cells foster reprogramming of BM adipocytes (BMAds) to secrete growth factors and pro-inflammatory adipokines, such as LEPTIN which can support MM survival and chemoresistance. The level of bone marrow tissue changes during the progression of MM disease. **(B)** Overview of potential of spheroids/scaffold 3D co-cultures, bone organoids, bone marrow organoids (BMOs), endosteal niche and combined bone and bone marrow (comBOs) to recapitulate myeloma bone and bone marrow disease. **(C)** Developmental workflow for personalized myeloma model with integrated bone marrow adipose tissue (BMAT) in organoid BMO/comBOs. Schematic representation of a patient-specific 3D BMO/comBO model incorporating bone marrow adipocytes (BMAds), multiple myeloma (MM) plasma cells, mesenchymal stromal cells, osteoclast precursors, osteoblast-lineage cells, and endothelial cells within a biomimetic scaffold or extracellular-matrix-based construct. Because patient-derived BMAds may be limited at different stages of MM, allogeneic BMAds or iPSC-derived cell phenotypes could provide an alternative. This model recapitulates key cellular, metabolic, paracrine, and bone-remodeling features of the MM bone niche for evaluating therapeutic sensitivity and resistance.

**Table 1:** Current achievements in modeling myeloma bone disease and integrated BMAT.

Evidence type	Main findings	System	Key limitations	Reference
Clinical/epidemiological	High BMI/obesity linked to higher MM risk and worse outcomes	Patient cohorts	Confounding; BMI not related to BMAT amounts	(19, 20)
Clinical imaging/histology	↑ fat fraction (21); ↓ trend in axial BMAT (22); ↓ BMAd density in progressing MGUS (23)	MRI, biopsies	Skeletal site heterogeneity; cross-sectional approach; small cohorts	(21–23)
Mechanistic, *in vivo*	Obesity accelerates MM; BMAT ↑ in early disease; reprogrammed BMAds drive bone disease	Mouse models	Species differences	(24–28)
Mechanistic, *in vitro*	Leptin, CCL2/CXCL12, exosomes support MM survival/chemoresistance; MM induces BMAd delipidation/SASP	Co-cultures	Cell lines; 2D; BMSC-derived (not native BMAds)	(29–35)
Established 3D models	MM cells engraft and retain phenotype in BMOs/comBOs	iPSC-based organoids	BMAds identified but models were not specifically adjusted for BMAT support	(36–38)
Proposed models	Patient-derived, site-specific, biomaterial-supported BMAT–MM models	iPSC-based organoids combined with patient-derived cells	Low amount of patient-derived BMAT; non- malignant patient-derived BMAT as alternative	This review

“↓” (decrease) and “↑” (increase).

## 2 The bone marrow niche in multiple myeloma: cellular components and extracellular matrix

The myeloma BM niche is a dynamic microenvironment whose cellular and non-cellular components actively fuel tumor survival, disease progression, and chemoresistance. This permissive niche comprises diverse cellular elements, including BMSCs, fibroblasts, osteoclasts, osteoblasts, endothelial cells and various immunosuppressive immune populations, all of which engage in extensive crosstalk with the extracellular matrix (ECM) (39, 40). Myeloma cells profoundly reshape this niche, establishing a self-reinforcing circuit driven by reciprocal signaling, ECM remodeling and inflammatory reprogramming that promotes immune evasion and therapeutic resistance. Importantly, the BM niche is highly plastic and undergoes continuous remodeling across disease stages, from MGUS to SMM, overt MM and relapse and/or refractory MM (RMM) (8, 41) (**Figure 1**). A deeper understanding of this dynamic interplay is essential for elucidating mechanisms of disease progression and identifying novel therapeutic strategies. Importantly, even though almost all patients with MM eventually experience RRMM, there is currently no established standard of care therapy for these patients (42). Bone environment mediated drug resistance is shown to contribute to minimal residual disease, protecting MM cells from eradication. In addition, bone lining cells promote osteoclast maturation in RRMM. Thus, the BM microenvironment in contributing to MM resistance and patient relapse. This provides a rationale for early targeting the BM microenvironment to achieve satisfying anti-myeloma effects (43).

Within this complex niche, BMSCs constitute a central stromal element and mediate many of the signals that support MM persistence. BMSCs support MM progression largely through paracrine signaling, releasing factors such as IL-6, CXCL12, IL-7, IL-8 and VEGF that promote myeloma cell survival, proliferation and chemoresistance (41). In addition, BMSCs interact with myeloma cells through adhesion molecules such as VLA-4, lymphocyte function-associated antigen 1 (LFA-1) and mucin 1 (MUC1), activating signaling pathways including NF-κB, MAPK, Notch and PI3K that enhance cell survival and proliferation (44). Yet another route of BMSC-mediated drug resistance includes exosomes which can transfer bioactive cargo to myeloma cells and promote survival though p38, p53, JNK and AKT, increased Bcl-2 and reduced caspase-9/caspase-3 cleavage or even increased proteasome activity contributing to proteasome inhibitor resistance (45–47). BMSCs also actively reprogram MM cells at epigenetic level, inducing chromatin remodeling and transcriptional changes that favor disease dissemination and early therapeutic resistance (48). Importantly, recent single-cell transcriptomic analysis of BMSCs in MM patients revealed their significant heterogeneity identifying the association of disease progression with decline in osteogenic BMSCs and expansion of immune-regulatory BMSCs, which contribute to bone destruction and immunosuppression (49). They also identified high mobility group (HMG)-high BMSC subpopulation, that appears to have strong regulatory activity and may potentially serve as risk stratification biomarker.

Notably, the decline of osteogenic BMSCs in MM coincides with a shift toward adipogenic commitment (18), pointing to BMAT as a further, increasingly recognized stromal component of the myeloma niche (**Figure 1A**). Under the influence of myeloma cells, BMAds are reprogrammed to secrete growth factors, adipokines and pro-inflammatory adipocytokines, which actively support disease progression (29, 30, 50). MM cells may also skew BMSC differentiation toward the adipogenic lineage, thereby increasing marrow adiposity. The resulting BMAds, in turn, promote myeloma cell proliferation, migration, and drug resistance through direct cell-cell contact and cytokine signaling, regulated by PPARγ-related pathways (31). Among adipokines involved, leptin has been shown to enhance MM growth, activate AKT and STAT3 signaling, increase Bcl-2, suppress caspase-3 and promote autophagy and chemoresistance (32, 33). Namely, higher BMAT level prior to treatment, protects MM cells against chemotherapy (high dose melphalan and dexamethasone followed by autologous bone marrow transplantation). Compared with non-irradiated BMAT, irradiation-induced BMAT senescence promoted a more dormant MM cell phenotype (30).

MM is characterized by immunosuppressive BM niche where immune cells are reprogrammed to support tumor tolerance, chronic inflammation and immune escape. Single-cell RNA sequencing analyses of BM samples from healthy donors, MGUS, SMM and overt MM show that immune dysfunction begins early, with altered NK-cell function, reduced cytotoxic T-cell surveillance and defective monocyte antigen presentation already present in precursor stages (51). Within established MM, T cells gradually lose antitumor activity, with expansion of Tregs and pro-inflammatory Th17 cells, while myeloma cells-derived IL-10 and decreased expression of IL-12p70 impair Th1 responses (52). At the same time, myeloid-derived suppressor cells, dysfunctional dendritic cells and tumor-associated macrophages promote immunosuppression, reduce antigen presentation and reinforce microenvironment that favors plasma-cell survival, migration and drug resistance mainly through IL-6, TGF-β, arginase and NF-kB (53–55). NK cells and cytotoxic T cells are also suppressed, further limiting immune-mediated clearance of myeloma cells (56). On the other hand, immune landscapes can be distinct in newly diagnosed MM and RRMM. It is shown that immune cells become dysfunctional following therapy whereas the MM clone may be less dependent on inflammation-mediated growth pathways and reduced sensitivity to IFN-mediated immunosurveillance (57). Insights into these differences in immune profiles in BM are of great importance for accurate MM modeling and development of therapy that overcomes chemoresistance.

Niche cells are embedded in a specialized ECM that further modulates myeloma cell behavior. Proteomic profiling revealed a markedly altered ECM and bone remodeling in BM of MM patients (**Figure 1A**), with upregulation of fibronectin, collagens I, III, and VI, laminins, and several proteoglycans compared with healthy BM (58). This remodel ECM promotes MM cell adhesion, survival, and proliferation. Conversely, decellularized ECM from normal bone reduced MM cell viability and increased chemosensitivity (59), whereas reduced elastin in MM BMSC-derived ECM promoted MM cell proliferation (60). ECM also shapes myeloid cell polarization. Biglycan drives pro-inflammatory TLR2/4 signaling, versican (VCAN) promotes immunosuppressive programs via TLR2/6, and its proteolytic fragment versikine induces an immunogenic, IRF8-dependent type I interferon and IL-12p40 response (61). Clinically, alterations in ECM-associated genes, such as VCAN and MUC16, carry prognostic significance in MM (62).

This complex microenvironment is poorly recapitulated by conventional two-dimensional (2D) cultures, which lack the adhesive, biomechanical, and biochemical cues of the native 3D BM niche that drive MM progression and drug resistance (**Figure 1B**). Consequently, monolayer cultures often yield altered cellular phenotypes, signaling, and drug responses. Compared with 2D cultures, 3D systems showed increased expression of cytokines and signaling molecules, such as SDF-1, IL-1α, TNF-α, MIP-1δ, and OPG, stronger drug resistance, and signs of MM cell de-differentiation, with lower CD38, CD56, and CD138 and higher CD34 expression (63). Similarly, 3D tissue-engineered bone models uncovered distinct miRNA signatures in myeloma cells that were not observed in 2D systems (64), while *ex vivo* 3D co-culture system, developed using cell-repopulated gelatin scaffolds and bioreactor system, showed stronger activation of pro-survival signaling, including pAKT, pSTAT and survivin, compared with their 2D counterparts (65). In addition, collagen-based 3D models of MM, where BMSCs and MM1.S cell line were used, showed reduced anti-myeloma effect of bortezomib (66). This clearly indicates relevance of 3D model for screening drug effects. However, simplified 3D models, such as spheroids obtained by co-culturing BMSCs and myeloma cell lines, are valuable baseline models, but their relevance in investigating myeloma disease might be limited. These findings highlight that 2D models provide incomplete or misleading insights into MM pathophysiology, underscoring the urgent need for more physiologically relevant 3D platforms to better elucidate mechanisms of pathogenesis and therapeutic resistance (**Figure 1B**). Improved accuracy at the preclinical stage will help lower rates of candidate compound failure at clinical trial stages.

## 3 Rationale for personalized modeling of myeloma

Longitudinal collection of bone marrow samples across the course of MM remains challenging, particularly during premalignant stages (67). This clearly limits understanding pathogenesis of MM disease. Patient material-based *ex vivo* 3D models can recapitulate key features of the tumor microenvironment and allow controlled, time-resolved assessment of treatment response (**Table 1**). By integrating these modeling-based outcomes along with clinical and molecular data, it could be possible to better capture patient-specific disease behavior and identify mechanisms of myeloma progression and resistance. In addition, these models would allow single-cell and multi-omics approaches to target myeloma heterogeneity (68), clonal evolution and improve early diagnosis and personalized treatment strategies (11, 69, 70). By linking whole-genome, exome, and RNA sequencing analyses with clinical data, the treatment could be related to a patient’s molecular subtype to avoid using a “one-size-fits-all strategy” (71). Importantly, MM drug sensitivity and resistance are not solely defined by tumor genetics, but also by functions of the BM microenvironment (72). Moreover, development of preclinical and patient-based 3D models that capture genetic heterogeneity of myeloma cells and immune remodeling in BM would provide next-generation platform to link MM genetics, immune context, and therapy response, and personalized therapy development (73) (**Figure 1C**).

## 4 Significance of bone marrow adipose tissue in myeloma disease

Clinical studies emphasized that being overweight and obesity are associated with worse outcomes in MM, with perspective to be modifiable in certain patient groups (19). Importantly, obesity may reduce MM immune surveillance which subsequently increases risk of developing MM (20, 24). Experimental data supports a mechanistic basis for this association: in the Vk*MYC mouse model, diet-induced obesity reduced marrow T and B cells and promoted tumor progression (24), and high-fat diet shifts BMSC differentiation from osteoblasts toward adipocytes (25, 26). Therefore, targeting fatty acid-binding proteins, involved in lipid transfer, has been suggested as promising strategy to combat obesity in MM (74).

There is reported positive association between diaphysis bone marrow fat fraction and MM (21). However, another study revealed a trend for lower axial bone marrow adiposity in MM patients (22). Importantly, recent study found bone marrow adipocyte (BMAd) density was decreased in MGUS patients who developed MM, compared to non-progressing MGUS patients (23). Therefore, BMAT-myeloma relationships may vary across skeletal sites and myeloma disease progression. In addition, for instance, patient-based evidence on BMAT specifically in relapsed MM is scarce. Our preliminary data showed reduced BMAT level in newly diagnosed MM patients when compared to non-malignant BM and relapsed MM patients. It should be noted that BM samples were obtained from different skeletal sites, namely the hip bone, iliac crest, and sternum. Accordingly, a larger cohort is required to assess whether BMAT alterations in MM patients are site-specific. As we previously revealed different lipid profiles of human BMAT from distinct skeletal sites (75), it is rational to hypothesize localization-specific changes of BMAT features in MM patients. Importantly, these clinical data are correlative and derive from heterogeneous, mostly cross-sectional cohorts that differ in skeletal site, assessment method (MRI fat fraction vs. histomorphometry), disease stage, and treatment history (**Table 1**). Age and BMI, both determinants of marrow adiposity, are additional potential confounders. Although Mendelian randomization approaches, such as those applied in the UK Biobank (21), offer a means to address causality, it currently remains unresolved whether altered BMAT is a cause or a consequence of MM, and even the direction of change (increased vs. decreased adiposity) is still inconsistent across studies.

Adipogenesis in BM is linked to the ability of BM to support MM cells, and in the main proportion of studies, it is BMSCs served as potential progenitors of osteoblast and BMAds to model BMAT-MM interactions and clarify BMAT relevance for MM. As it has been shown that adipogenic BMSCs cannot fully recapitulate BMAd profile and cholesterol metabolism (76), it is not surprising that we still search for the better model to deconvolute the role of BMAT in MM disease. Recent *ex vivo* profiling study in MM showed that therapeutic sensitivity is shaped not only by tumor-intrinsic genetics but also by BM microenvironmental features, underscoring the need for advanced 3D models that incorporate niche components such as BMAT (72).

In contrast to clinical studies, mechanistic insights into BMAT–MM interactions derive almost exclusively from murine models and *in vitro* co-cultures (**Table 1**). Current findings suggest that BMAT and BMAds stimulate MM disease, where MM can reprogram BMAd into MM-supportive cells (27, 30, 77). Using of Randl mice model revealed increased amount of BMAT during the early stage of myeloma disease, where BMAds stimulated myeloma cell growth and survival (28, 72). In addition, the latest data indicates that BMAd-derived factors like CCL2 and CXCL12 have chemotactic roles that support MM cells via BMAds (29, 34). Recent study reported pro-survival effects of BMAd-derived exosomes obtained from MM patients on myeloma cells exposed to bortezomib (35). Importantly, it has been reported that adipogenic potential of BMSCs from MM patients is reduced when compared to cells from healthy donor BMSCs (72), which is of particular importance for BMSCs incorporation in complex 3D models. Conversely, BMAT actively regulates bone remodeling. PPARγ drives adipogenesis at the expense of osteoblastogenesis and is antagonized by Wnt/β-catenin signaling (78), a balance that MM shifts toward adipogenesis via PPARγ2 stabilization, DKK1, and sclerostin (6, 7, 17, 18). BMAds and their precursors, in turn, promote bone resorption through RANKL expression (79) and inflammatory cytokines, such as IL-6 and TNF-α (27, 30), establishing a self-reinforcing cycle between myeloma-driven adipogenesis, impaired bone formation, and enhanced resorption (**Figure 1A**).

BMAT–MM interactions also converge on mTOR complex 1 (mTORC1), a conserved nutrient sensor integrating growth factor, energy, and amino acid signals to control protein synthesis and cell growth (80, 81). This is particularly relevant in MM, given the high protein synthesis demand and glutamine addiction of myeloma cells (82). BMAds supply fatty acids to MM cells (36) and modulate this pathway via adipokines: leptin and resistin activate AKT (32), whereas adiponectin, suppressed by MM cells, activates AMPK, a negative regulator of mTORC1 (37). Collectively, these findings implicate strong impact of BMAT on myeloma disease, and therefore further incorporation of BMAds and/or their secreted factors into *in vitro* and *ex vivo* models are important. However, most of these studies relied on murine BMAds or BMSC-derived adipocytes, myeloma cell lines, and 2D culture conditions, which do not fully reflect the biology of human BMAds (76). Therefore, while these data provide plausible mechanisms, their contribution to disease in patients remains to be validated.

## 5 3D bone marrow adipose tissue modeling

According to the discrepancies between clinical and experimental findings, there are several questions that current approaches cannot but should resolve: whether BMAT changes precede or follow progression from MGUS to MM (23); whether BMAT–MM interactions differ across skeletal sites (21, 22, 75); whether pro-tumorigenic BMAd functions identified in mice are conserved in human BMAds; and how BMAT contributes to drug resistance and relapse (30, 35). Addressing these questions requires human, patient-derived 3D systems, as discussed below. Here, we distinguish between 3D models that have already been applied to MM, such as BM organoids engrafted with patient-derived myeloma cells (38, 83–89), and approaches that have not yet been combined with BMAT or myeloma cells and therefore represent proposed strategies. Successful modeling of BMAT requires knowledge of the physiology and structure of the bone marrow and adipose tissue. Namely, BMAT is a functional compartment integrating vascular, immune, ECM, and metabolic components, which should be reproduced in experimental models. First, human BMAds exhibit a distinct, cholesterol-oriented lipid metabolism and site-specific lipid profiles (75, 76), which myeloma cells exploit by inducing lipolysis and fatty acid uptake (88); because BMSC-derived pre-adipocytes only partially recapitulate these features (76), primary BMAds represent a valuable alternative. Also, models should reproduce the BMAT secretome, including adipokines such as adiponectin, which is suppressed by MM cells (28), and leptin, as well as inflammatory and chemotactic factors such as SASP components and CCL2/CXCL12 (29, 30, 32). Interactions with macrophages and T cells are also required to assess the contribution of BMAT to immunosuppression. In addition, the BM is physiologically hypoxic (~1–4% O_2_) (90), which regulates both adipogenesis and MM behavior, making physioxic culture, as applied in comBOs (87), essential. Importantly, biomaterials should mimic the soft marrow matrix and adipocyte-associated ECM components, such as collagens IV and VI and laminin (58, 83), since matrix stiffness directs BMSC lineage commitment (91). Finally, BMAT is closely associated with sinusoidal vessels and perivascular adipogenic progenitors, including non-lipid-laden marrow adipogenic lineage precursors (92), which should be incorporated through endothelial co-culture or iPSC-derived organoids (87, 88). No current model recapitulates all these features, and they should serve as validation criteria for next-generation BMAT–MM models (**Table 1**).

By preserving multicellular interactions, ECM architecture, and key biophysical features of physiological and diseased BM microenvironment, 3D systems enable more physiologically relevant investigation of myeloma cell survival, stromal support, treatment resistance, and disease-associated bone remodeling. Within this framework, bone marrow organoid (BMO) models (38) (**Figure 1C**) are particularly suited to capturing tumor/hematopoietic-stromal interactions, whereas bone organoids (83) more directly model osteogenic differentiation, mineralized matrix formation, and the dynamic balance between bone formation and resorption. Compared with spheroids, these complementary organoid platforms better recapitulate tissue architecture, cellular heterogeneity, and long-term culture, providing a basis for dissecting myeloma bone disease and testing therapies that target both malignant PCs and the skeletal microenvironment.

Biofabrication of 3D bone organoids for modeling skeletal organogenesis, development and disease relies on cell-based, scaffold-free and scaffold-based approaches (83). Cell-based bone organoids rely on the self-organizing capacity of skeletal cells and are suited to studying development, mineralization, and osteogenesis-modulating drugs, whereas scaffold-based organoids use natural or synthetic biomaterials to guide cellular organization and differentiation (83).

In the previous study, human BMSCs were organized into a functional 3D self-organizing co-culture of osteoblasts and osteocytes, creating an organoid for early-stage bone (woven bone) formation (84). In addition, a trabecular bone organoid model (85) showed cellular activities and the effect of spatiotemporal profiles of regulatory molecules on bone remodeling activity. By using bovine bone blocks, murine chip cultures and BM mononuclear cells, this bone model successfully recapitulated coexisting active and resting bone surfaces. Another study reported a scaffold- and bioreactor-free method of creating osteochondral organoids *in vitro* based on stimulated chondrogenic and osteogenic differentiation in induced pluripotent stem cells (iPSCs), which recapitulates endochondral ossification. Importantly, this study revealed that formed 3D cartilaginous matrix prevented reinduction of pluripotency in iPSCs (86). This finding is of great importance for utilization of iPSCs in organoid fabrication. A recent study revealed the combined bone and lympho-myeloid bone marrow organoid (comBOs), iPSC-derived model that generates osteolineage, vascular, lymphoid, and myeloid compartments within a single organoid under physioxia (5% O_2_) (87). ComBOs showed capacity to sustain long-term lympho-myeloid potential in serial organoid re-seeding assays. BMOs and comBOs support the engraftment, survival, and proliferation of cells from patients with myeloid and lymphoid hematologic malignancies including MM, acute lymphoblastic leukemia (ALL), and myeloid leukemias, and CD34+ cells of patients with myelodysplastic syndrome (MDS) (87–89). Engrafted patient cells preserved their genetic profiles during proliferation and differentiation in BMOs. These BMOs were obtained by iPSC culturing in ultra-low attachment plate to form embryo bodies, which were embedded in hydrogel containing Collagen IV. Other bone marrow subsets, including endothelial cells, neutrophils, monocytes, lymphocytes, and adipocytes, were also detected by immunolabeling (93). Endosteal niche models, which reconstruct the bone–marrow interface (83) represent an important advance for MM research because they recreate key human BM features, including vascularized osteogenic compartments and niche-derived signaling pathways that regulate hematopoietic and malignant cell behavior. Indeed, patient-derived myeloma cells expanded and remained viable for more than 12 days after engraftment into a BMO. In addition, patient cells retained their original immunophenotype (CD38^+^CD319^+^CD56^+^) more successfully in the organoids than in the 3D BMSC spheroids (88). Additionally, comBOs recapitulated myeloma BM niche remodeling and identify MIF signaling as triggering disease factors. This study confirmed a lumen-forming, CD34^+^ vascular network, CD271^+^ perivascular cells, Bodipy^+^ cells (adipogenesis-related) and osteocalcin deposition (early osteogenesis) in generated comBOs (87). Collectively, it appears that advanced multicellular models based on stem cells represent perspective players in future creation of MM models with BMAT. However, full recapitulation of BMAT requires additional knowledge on adipocyte biology and adipose tissue models.

Modeling adipose tissue in different depots [subcutaneous (94), visceral (95), mammary (96), brown (97)] has contributed to the understanding of adipose tissue physiology and its important role in pathologies, such as obesity, cancer and atrial dysfunction. Also, the results of these studies have shown site-specific differences among adipose tissues, but at this level there are still no findings when it comes to BMAT. These *in vitro* non-animal models (NAMs) are new methodology approaches, are capable of recapitulating adipocyte physiology and tissue pathology, and combining them with high-throughput downstream assays bring new possibilities in precise and personalized therapy of diseases. A recent study reported adipose 3D model recapitulating obese phenotype. The model included adipose tissue dysfunctions, including adipocyte hypertrophy, ectopic lipid accumulation, low-grade inflammation (hypoxia and fibrosis), and insulin resistance (98, 99).

Although murine adipose models continue to be extensively used in research studies, they do not accurately replicate human physiology and/or pathology. Also, the costs associated with animal models, together with ethical considerations and technical feasibility, have further encouraged researchers to develop and employ sophisticated *in vitro* 3D models. The 3D adipose tissue models are either scaffold-free (spheroids and organoids) or scaffold-based. Spheroids obtained by culturing human adipose stromal vascular fraction cells represent the second most commonly used method in modelling human adipose tissue and cancer interaction among the studies analyzed (100).

In addition, there is BMAT 3D model created using a silk fibroin scaffold, with mouse BMSCs differentiated toward adipocytes, and the addition of myeloma cell lines. Importantly, this study showed MM cells contributed to adipogenic BMSCs delipidation. Thus, it has been concluded that myeloma cells are involved in BMAT level regulation in myeloma patients (72). Another study utilized reconstituted basement membrane DQ-Collagen IV substrate to establish 3D model of BMAT, by using differentiated BMSCs and combining them with prostate cancer cells (101). Combining this knowledge and understanding other adipose tissue 3D strategies, it can be concluded that cell-based compartment should be supported with biomaterial to biofabricate BMAT (**Figure 1C**).

## 6 Conclusion

Experience gained from 3D adipose tissue modeling provides tremendous knowledge for development next-generation BMAT models. These strategies, including scaffold design, cellular composition, and dynamic culture conditions, can be adapted to better recapitulate the unique structural and functional properties of BMAT. Leveraging this knowledge will accelerate the development of physiologically relevant BMAT 3D models, enabling more accurate investigation of their role in bone biology, hematological malignancies, and metabolic regulation. Current clinical data establishes associations between adiposity and MM but not causality, whereas mechanistic evidence comes predominantly from murine and simplified *in vitro* systems. Thus, patient-derived 3D BMAT models are proposed here to connect these two levels of evidence. Moreover, such improvements are expected to enhance the translational relevance of *in vitro* findings, bridging the gap between experimental systems and clinical applications.
